# Acute Bronchiolitis: Is There a Role for Lung Ultrasound?

**DOI:** 10.3390/diagnostics9040172

**Published:** 2019-11-01

**Authors:** Antonio Di Mauro, Angela Ammirabile, Michele Quercia, Raffaella Panza, Manuela Capozza, Mariano M. Manzionna, Nicola Laforgia

**Affiliations:** 1 Department of Biomedical Science and Human Oncology, “Aldo Moro” University of Bari, 70100 Bari, Italy; michelequercia@libero.it (M.Q.); raffaella.panza@uniba.it (R.P.); manuelacapozza26@gmail.com (M.C.); nicola.laforgia@uniba.it (N.L.); 2 Unità Operativa Complessa, Pediatric and Neonatology, San Paolo Hospital, ASL BARI, 70100 Bari, Italy; mariano.manzionna@alice.it

**Keywords:** Ultrasonography [Mesh], bronchiolitis [Mesh], bronchiolitis, Viral [Mesh], lung ultrasound

## Abstract

Introduction: Viral bronchiolitis is a common cause of lower respiratory tract infection in the first year of life, considered a health burden because of its morbidity and costs. Its diagnosis is based on history and physical examination and the role of radiographic examination is limited to atypical cases. Thus far, Lung Ultrasound (LUS) is not considered in the diagnostic algorithm for bronchiolitis. Methods: PubMed database was searched for trials reporting on lung ultrasound examination and involving infants with a diagnosis of bronchiolitis. Results: Eight studies were suitable. Conclusions: This review analyzed the current evidence about the potential usefulness of LUS in the clinical management of bronchiolitis. Literature supports a peculiar role of LUS in the evaluation of the affected children, considering it as a reliable imaging test that could benefit the clinical management of bronchiolitis.

## 1. Introduction

Bronchiolitis is a lower respiratory tract infection (LRTI), resulting from the inhalation of virus-containing droplets. The most common cause is the Respiratory Syncytial Virus (RSV), identified in 50–80% of patients, and responsible for the most severe infection.

Bronchiolitis usually occurs during the first year of life, with a peak incidence between 3 and 6 months of age, and represents the main reason for hospitalization in the young population [1].

The diagnosis of bronchiolitis is based on the history and the physical examination [2]. Viral testing, bacterial cultures, complete blood count and blood gas, pulse oximetry and chest radiography are not routinely included in the diagnostic workup.

In the last decades, Lung Ultrasound (LUS) has been proposed as a diagnostic tool in many diseases in the pediatric and neonatological settings [3,4,5]. In particular, the anatomical conformation of children makes the sonographic evaluation of the lungs possible for the creation of acoustic windows by the relatively unossified thorax of children, the large thymus gland and the thinner subcutaneous tissue [6].

Currently, LUS is not considered in the diagnostic algorithm for bronchiolitis, even though its usefulness has been tested in several studies in the last years as an emerging diagnostic tool. Its main benefits are the absence of the irradiation risk, the possibility of a bedside evaluation, and the short time needed for a report.

In this review, we analyze the current evidence about the potential usefulness of LUS in the clinical management of bronchiolitis.

## 2. Methods

An exhaustive search for eligible studies has been performed, using the PubMed database as data source. The following subject MeSH headings were used: “Ultrasonography”[Mesh], “Bronchiolitis”[Mesh], “Bronchiolitis, Viral”[Mesh], “Lung”[Mesh], “Infant”[Mesh]. Furthermore, free text and proper Boolean operators “AND” “OR” were also included to be as comprehensive as possible. Additional studies were sought using references in articles retrieved from searches.

A.D.M. and M.M. screened titles and abstracts from the database, removing duplicates, and analyzed the full-text version of the selected studies to assess their eligibility. All the chosen articles have been published in the last eight years, starting from 2011, when the first study about the use of LUS in acute bronchiolitis was performed. The exclusion criteria were: articles not related to the field of interest, studies without relevant conclusions, specific types of articles (case reports, editorials or letters, comments, review articles). Search limits were set for trial involving only human subjects.

Moreover, this review has been conducted according to the Preferred Reporting Items for Systematic Review and Meta-Analyses (PRISMA) Guidelines to ensure a transparent reporting of a systematic review [7].

A systematic approach for the data extraction has been applied to each considered study: main features (language, authors, type of study, year of publication, purpose), methods (study population), clinical and/or radiographic examination, sonographic examination (bronchiolitis-specific LUS findings), results about LUS diagnostic usefulness considering primary and potentially secondary outcomes (including sensitivity, specificity, positive and negative predictive values, accuracy).

## 3. LUS in Management of Bronchiolitis

We found eight trials reporting on lung ultrasound examination and involving infants with a diagnosis of bronchiolitis (Table 1).

### 3.1. LUS Findings in Bronchiolitis

The first study investigating the usefulness of LUS as a potential tool for the diagnosis of bronchiolitis was performed by Caiulo et al. in 52 young children under 16 months of age affected by bronchiolitis [8]. They aimed to describe the LUS findings in children with bronchiolitis and to compare the diagnostic accuracy of LUS and CXR.

They underwent an LUS examination on the same day of the CXR, and the LUS follow-up was stopped only after a complete normalization of lung appearance that occurred between day 10 and day 12 in all the affected children. The severity of the disease was assessed by a modified version of Downes’ Score, one of the main scoring systems for respiratory distress in newborns, based on five parameters: PO2 (the partial pressure of oxygen usually decreases due to airways obstruction), inspiratory breath sounds, use of accessory muscles, presence of expiratory wheezing and respiratory rate. Each criterion was rated on a scale from 0 to 2, and then, a total score was calculated. LUS findings varied according to the disease severity: a prevalence of few isolate B-lines in the mildest form, a higher frequency of single subpleural consolidation and areas of white lung in the moderate disease, the presence of multiple lung consolidations, pleural line anomalies and pleural effusions in the most severe ones. In all cases, the described signs completely disappeared during the monitoring of patients.

Furthermore, they noted that LUS was able to identify lung abnormalities not revealed by CXR, such as minimal pleural effusion and small pneumothorax. In particular, nine patients with a course of bronchiolitis had normal CXR and abnormal LUS. They sustained a higher reliability of LUS compared to CXR for diagnosis and follow-up of bronchiolitis and a lower time in execution of the test.

Although guidelines limit the use of CXR for the diagnosis of bronchiolitis only to atypical presentations (toxic appearance, bacterial superinfection or pneumothorax as complications) [2] and British Thoracic Society (BTS) guidelines [16] do not consider CXR as a routine test in the management of community-acquired pneumonia, CXR is performed in around 50% of bronchiolitis cases, with the main goal to rule out pneumonia. Apart from the danger related to the exposure of children to ionizing radiations, the use of CXR in bronchiolitis management has been associated with other limitations: the increased prescription of useless antibiotics for the similar radiographic appearance of consolidation and atelectasis, the impossibility to distinguish bacterial and viral pneumonia, variable inter-observer interpretation, high medical costs and longer time [17].

Other authors compared findings at LUS and CXR of children with RSV-associated bronchiolitis through a retrospective study. Jaszczolt et al. [9] underlined the utility of LUS again, considering safety, short time of examination, high sensitivity in finding pleural effusion, small consolidations and signs of interstitial infiltrations. On one hand, the classification of LUS findings included liver-like hypoechoic consolidations (small subpleural < 10 mm in diameter and > 10 mm in diameter with air bronchogram), interstitial syndrome with B7 lines (7 mm between two different lines), alveolar-interstitial syndrome with B3 lines (distance around 3 mm between two different lines), pleural effusion. On the other hand, the considered CXR anomalies were inflammatory infiltrate, hilar enlargement, peribronchial cuffing, pleural effusion and lung hyperexpansion. The most common findings of bronchiolitis at LUS were alveolar-interstitial syndromes and medium-sized subpleural consolidations with air bronchogram with maximum diameter of 30 mm, especially in the inferior and posterior areas of the lungs. The main result was that LUS may be a useful tool in the diagnosis of bronchiolitis for its high sensitivity in finding pleural effusion, small consolidations and signs of interstitial infiltrations. Finally, they concluded that a direct comparison of both methods was not possible, considering the greater ability of CXR in showing bronchi and hilar regions or lung hyperinflation.

### 3.2. LUS and Clinical Progression of Bronchiolitis

Another group of studies demonstrate the possibility to predict the clinical progression of bronchiolitis through different proposed ultrasound scores.

Basile et al. [10] performed an observation cohort study in 106 infants with bronchiolitis, diagnosed with physical examination and lung ultrasound scans. The severity of the disease was described according to a clinical score (tachypnoea, dyspnoea, use of accessory muscles and breath sound) and a sonographic score (presence of B-lines, subpleural lung consolidations, bilateral involvement of intercostal spaces): a high agreement of 90.6% was demonstrated between the two scores, a further demonstration of the accuracy of LUS in the diagnosis and management of bronchiolitis in children. In addition, authors discovered that LUS was able to identify children in need of supplementary oxygen with a very high specificity (98.7%) and sensitivity (96.6%), considering as main indexes subpleural lung consolidation > 1 cm in the posterior area or a severe interstitial syndrome according to the number of involved bilateral intercostal spaces. They also found an excellent inter-observer ultrasound concordance between diagnoses made by different sonographers.

Zoido Garrote et al. [11] confirmed the possibility to predict the clinical progression of bronchiolitis, using different clinical and ultrasound scores. This observational prospective study determined the presence of a moderate correlation between findings at early LUS (at the time of admission) and the severity of acute bronchiolitis, also taking its clinical progression into consideration. The main research areas were the need of Intensive Care Unit admission, the use of Invasive Ventilation, the length of hospitalization and the duration of oxygen therapy. As previously discussed, the main LUS findings associated to bronchiolitis were pleural line anomalies (PAs) with thickening and loss of lung sliding, extent of interstitial syndrome (AIS) as shown by B-lines up to the white lung appearance, the presence of subpleural consolidations (SCs) with a classification according to their number and their dimension. The total LUS score was in the range of 0–50 points, resulting from the sum of the scores for each individual zone (five areas: anterior parasternal, anterior axillary, posterior axillary, posterior paravertebral, posterior linea scapularis). The authors concluded that their results were consistent with the ones of the aforementioned studies [8,12] regarding the correlation with the clinical severity scores, and they confirmed the presence of a higher percentage of pulmonary anomalies in the posterior paravertebral and subscapular area, probably due to gravity or to the obligate supine position of the child.

An additional prospective, multicenter research trial has demonstrated that LUS could be a helpful tool to identify patients at risk for any type of respiratory support (non-invasive or invasive ventilation, except conventional low-flow oxygen through nasal sprongs) in the acute phase of bronchiolitis, starting from the idea that clinical scores have proven to be inaccurate in predicting prognosis. In fact, Bueno-Campaña et al. [12] have analyzed the data of 145 infants < 6 months with the aim to build a score based on LUS findings (presence and localization of B-lines, B-lines confluence and/or consolidation) performed at admission, but also on clinical data (age < or > 1 month and Wood-Downes-Ferres Score, typically used for asthma). Their results were consistent with the ones of the previously described studies, especially regarding the increased association of NIV with posterior consolidations > 1 cm. Moreover, it was the first study to explore LUS usefulness as a predictive tool for the need of respiratory support other than supplemental oxygen in acute bronchiolitis [12].

Another recent paper published in 2018 examined the use of LUS in infants admitted in the Paediatric ICU for severe acute bronchiolitis. This prospective observational single-center study was conducted in France by Taveira et al. [13] to evaluate the correlation between a quantitative LUS score and the length of Non-Invasive Ventilation (NIV) in 47 children under 6 months of age with severe bronchiolitis. The severity of the LUS-diagnosed lesions was considered for each of the six quadrants the lung was divided in, according to a score in the range of 0–2 points (total range 0–24 points): 0 for a healthy lung or a mild interstitial-syndrome with few B-lines, 1 for a severe interstitial syndrome with compact B-lines and with lung appearance, 2 for the presence of consolidations seen as a hypoechoic area with blurred margins and air bronchograms. Moreover, the number of involved intercostal spaces was defined with a horizontal scan. The results of the study did not confirm the primary aim to predict NIV length (CPAP or high-flow nasal cannula), nor an association with length of hospitalization or the clinical score mWCAS. The only statistically significant correlation was between the number of affected intercostal spaces on the right side (white lung appearance, typical of bronchiolitis) and the length on the oxygen therapy (days), which was considered a secondary endpoint [13].

### 3.3. Differential Diagnosis and Complications

Finally, two studies concern the role of LUS in the differential diagnosis of bronchiolitis or in the evaluation of its complications. Varshney et al. [14] conducted a prospective cross-sectional study to describe how LUS could represent a useful tool in a Pediatric Emergency Department (PED) for the assessment of patients with respiratory distress, signs of respiratory tract infection (rhinorrhea and/or cough) and concomitant wheeze. In fact, this clinical presentation can be suggestive of three pathologies that require different types of treatment: bronchiolitis, which needs only supportive care, asthma, which requires bronchodilators, and pneumonia, which improves with antibiotics. Obviously, a CXR can be useful for the diagnosis of pneumonia, but it increases the risk of unnecessary antibiotic administration in children affected by bronchiolitis. A key point of the study was the definition of a positive LUS and the related diagnosis. An LUS was considered pathological if one or more findings occurred in any of the lung zones: more than three B-lines per intercostal space, consolidation, pleural line abnormalities or fluid. On the other hand, a negative LUS was characterized by the absence of aforementioned findings and the presence of A-lines and lung sliding. The important contribution of this study to the literature concerns the possibility to rule out the diagnosis of asthma in case of positive LUS that could direct the clinician toward a diagnosis of pneumonia (100% of patients) or bronchiolitis (46% of patients) [14].

At last, Biagi et al. [15] tried to assess the diagnostic accuracy and reliability of LUS in children with a clinical suspicion of secondary bacterial pneumonia. LUS was performed 12 h after a posteroanterior CXR (lateral projection is not recommended to prevent further radiation according to BTS guidelines), according to the division of each hemithorax in the three different regions. The criteria for a definite diagnosis of pneumonia on LUS were a hypoechoic area with blurred margins and decreased echogenicity of the pleural line, the presence of B-lines and dynamic bronchograms, and the absence of lung sliding. Furthermore, the typical findings of simple bronchiolitis were small subpleural consolidations, absence of air bronchograms, pleural line abnormalities, single or confluent B lines, which could be associated also with viral pneumonia. Two thirds of patients showed consolidation in the posterior lung areas. The authors deduced that LUS has a higher sensitivity for the diagnosis of pneumonia than CXR (100% versus 96%) and its specificity can reach 98.4% when only consolidations >1 cm are considered [15].

## 4. Discussion

Traditionally, pulmonary pathologies have been studied with CXR and the first description of LUS as alternative diagnostic imaging test was published in 2012 in the “International evidence-based recommendations for point-of-care lung ultrasound”. This report defines LUS application in the diagnosis of pneumothorax, pleural effusion and lung consolidation, according to the literature of the previous 20 years. [18] However, the thought of LUS as a potential tool for the diagnosis of bronchiolitis is much more recent, since the first study about this topic was performed in 2011 by Caiulo et al.; they concluded that LUS could become the routine imaging modality for this group of patients. This data has been confirmed in successive studies, evaluating the association between clinical and US findings, generally through the use of scores, and the agreement between LUS and CXR. The main results were about a high reliability of LUS, considering a strong clinical-sonographic correlation, safety, short time of examination, high sensitivity in finding pleural effusion, small consolidations and signs of interstitial infiltrations. In fact, they underline the role of LUS to diagnose bronchiolitis and rule out primary pneumonia through specific findings, such as B-lines, white lung appearance and pleural line anomalies, or to diagnose secondary pneumonia as a complication, considering the peculiar presence of dynamic bronchograms and the absence of lung sliding.

We acknowledge some limitations of our study. The selected studies demonstrate a heterogeneity regarding the methods, the clinical and US scores and the considered outcomes. For this reason, the comparison does not allow definitive conclusions. However, good results have already been achieved considering the reliability of LUS in the management of bronchiolitis. In the future, as the number of trials regarding this imaging test grow, a single standardized US score could be validated and internationally used.

## 5. Conclusions

In summary, LUS can be considered a safe, non-invasive, quick, reproducible, low-cost, real-time imaging test that could benefit the clinical management of bronchiolitis: the interpretation of artifacts generated at the pleural surface in a bronchiolitis-specific pattern (presence of B-lines, subpleural lung consolidations, bilateral involvement of intercostal spaces) strictly correlates with the clinical evaluations in infants with bronchiolitis. A key point is that the routine use of LUS for children with bronchiolitis could reduce the number of performed CXR, the exposure to ionizing radiations with its dangerous consequences in children, i.e., the increased risk of cancer, and could avoid the useless administration of antibiotics. Future prospective studies may elucidate whether LUS can be used to help guide diagnostic and therapeutic decisions for affected children such as need for admission, oxygen therapy, ventilation and possibly to confirm the safety of outpatient management.

## Figures and Tables

**Table 1:** Summary of the analyzed studies about the role of Lung Ultrasound (LUS) in the management of bronchiolitis.

Authors	Type of study	Number of cases	LUS pattern	Results	Conclusions
Caiulo et al [8].	Prospective observational study	52 patients with bronchiolitis who had undergone a CXR for clinical reasons, median age 2.1 months	- Mildest form: a prevalence of few isolate B-lines - Moderate disease: a higher frequency of single subpleural consolidation and areas of white lung - Severe disease: the presence of multiple lung consolidations, pleural line anomalies and pleural effusions	- Positive tests for diagnosis of bronchiolitis: 47/52 for LUS vs. 38/52 for CXR. 9 patients with a clinical course of bronchiolitis had with normal CXR and abnormal LUS. - Mean time of tests interpretation: 0 m (during the scan) for LUS vs. 4.45 h for CXR. - Statistically significant LUS findings (*p* < 0.001 vs control group): lung consolidations (44/52 infants), numerous compact B-lines (34/52 infants), pleural line abnormalities (23/52 infants). - Not statistically significant LUS findings (*p* = ns 0.001 vs control group): few isolated B-lines (5/52 infants), considered as a normal finding. - LUS findings not revealed by CXR: minimal pleural effusion (3/52 infants), small pneumothorax (1/52 infants)	- Higher reliability of LUS for diagnosis and follow-up of bronchiolitis, due to identification of lung abnormalities not revealed by CXR
Jaszczolt et al [9].	Retrospective study	23 children aged 2 weeks to 24 months and 3 children older than 24 months with confirmed respiratory syncytial virus infection plus a separate specific group composed by 3 children over the age of 2 years also diagnosed with bronchiolitis	- Liver-like hypoechoic consolidations (small subpleural < 10 mm in diameter and > 10 mm in diameter with air bronchogram), Interstitial syndrome with B7 lines (7mm between two different lines), Alveolar-interstitial syndrome with B3 lines (distance around 3mm between two different lines), Pleural effusion - Higher involvement of inferior and posterior areas	- Positive tests for diagnosis of bronchiolitis: 21/26 for LUS vs. 4/26 for CXR. 5 children showed absence of abnormalities at LUS and CXR. - Statistically significant LUS findings (*p* < 0.001 vs control group): inflammatory consolidations > 10 mm (11/26 infants), small consolidations < 10 mm (8/26 infants), interstitial syndromes (6/26 infants), alveolar interstitial syndromes (11/26 infants), small amount of pleural effusion (3/26 infants).	- LUS as a useful tool in the diagnosis of bronchiolitis, considering its superiority in the detection of small amounts of pleural effusions or intraparenchymal lesions, and also in the monitoring of treatment efficacy. - Greater ability of CXR in showing bronchi and hilar regions or lung hyperinflation. - Direct comparison of specificity and sensitivity of both methods is not possible
Basile et al [10].	Observational cohort study	106 infants, median age of 71 days: 74 infants with mild bronchiolitis, 30 infants with moderate bronchiolitis, 2 infants with severe bronchiolitis	- Presence of B-lines, subpleural lung consolidations, bilateral involvement of intercostal spaces - Higher involvement of posterior and paravertebral areas	- High agreement between the attending physician and the paediatric sonographer on the severity of bronchiolitis (agreement: 90.6%; expected agreement: 52.3%; K = 0.8; Standard error = 0.0765; z = 10.19; *p* = 0.000). - Excellent inter-observer concordance on the basis of the US findings between the two different sonographers (Cohen’s kappa coefficient: agreement = 89.6%; expected agreement 46.4%; K = 0.8; Std error = 0.07; z = 11.33; *p* = 0.000). - LUS identification of those infants in need of supplementary oxygen with a specificity of 98.7% (95% CI: 93% to 99.8%), a sensitivity of 96.6% (95% CI: 82.2% to 99.4%), a positive predictive value of 96.6% (95% CI: 82.2% to 99.4%) and a negative predictive value of 98.7% (95% CI: 92.95% to 99.8%): True Positive (TP) as LUS score > 3 indicating the need of supplementary oxygen.	- High agreement (90.6%) between the clinical and sonographic scores and an excellent inter-observer ultrasound diagnosis concordance - Identification of children in need of supplementary oxygen with a very high specificity (98.7%) and sensitivity (96.6%)
Zoido Garrote et al [11].	Prospective observational study	59 patients with mild-to-moderate AB, median age of 90 days	- Pleural line anomalies with thickening and loss of lung sliding, extent of interstitial syndrome as shown by B-lines up to the white lung appearance, the presence of subpleural consolidations with a classification according to their number and their dimension.	- Moderate linear association between the sonographic score, especially the degree of AIS and SCs, and the clinical scores—modified WDFM (rho = 0.504, *p* < 0.001) and HSJD (rho = 0.518; *p* < 0.001), all clinical parameters except the respiratory rate. - High interrater intraclass correlation coefficient (absolute agreement between individual measurements) for the total score in the LUS-Sc (agreement = 0.917, 95% CI, 0.854–0.956). - Association between the sonographic score and the admission to PICU (OR 2.5; 95% CI: 1.1–5.9; *p* = 0.035), longer hospital stay (1.2 days; 95% CI: 0.55–1.86; *p* = 0.001) and duration of oxygen therapy (0.87 days; 95% CI: 0.26–1.48; *p* = 0.006). - Association between each 5-point increase in the LUS-Sc and increase in the probability of admission to PICU (OR, 2.5; 95% CI, 1.1–5.9; *p* = 0.035), in length of stay by 1.2 days (95% CI, 0.55–1.86; *p* < 0.001) and in duration of oxygen therapy by 0.87 days (95%CI, 0.26–1.48; *p* = 0.006).	- Moderate correlation between findings at early LUS (at the time of admission) and the severity of acute bronchiolitis, taking also its clinical progression into consideration
Bueno-Campaña et al [12].	Prospective multicentre study	145 infants with acute bronchiolitis, median age of 1.7 months	- Lung sliding, ≥3 B-lines per intercostal space (uni or bilaterally located), confluent B-lines (uni or bilaterally located), and subpleural consolidations (larger or smaller than 1 cm).	- Multivariate final predictive model: age less than 1 month (1.5 point), WDFS ≥ 6 points (2.5 points), more than 3 B lines per intercostal space (1.5 point) and confluent B lines bilaterally in anterior area (1 point) and posterior consolidations of any size (1 point if < 1 cm and 3 points if > 1 cm). - Predictive capacity for the need of respiratory support of a cut-off point of 3.5: sensitivity of 89.1% (CI95%:78.2–94.9%), specificity of 56% (CI95%: 45.3–66.1%), PPV of 57% (CI95%:46.4–66.9%), and NPV of 88.7% (CI95%:77.4–94.7) with an area under ROC: 0.845 (CI95%:0.781–0.909). - Statistically significant correlation (*p* < 0.0001) between the predictive score and length of stay (rho = 0.401), and duration of Oxygen-therapy (rho = 0.448).	- Presence of at least one posterior consolidation > 1 cm as main factor associated to the need of respiratory support (non-invasive or invasive ventilation, except conventional low-flow oxygen through nasal sprongs) in the acute phase of bronchiolitis
Taveira et al [13].	Prospective observational single-center study	47 infants under 6 months of age with severe acute viral bronchiolitis	- Mild disease: mild interstitial-syndrome with few B-lines - Moderate disease: severe interstitial syndrome with compact B-lines and with lung appearance - Severe disease: presence of consolidations seen as a hypoechoic area with blurred margins and air bronchograms	- Absence of statistically significant correlation between the LUS score on admission (3.5 ± 2.6) and length of NIV (69 ± 68.6 hours, rho = 0.1, *p* 0.51), mWCAS score (4 ± 1.6, rho = 0.09, *p* 0.57), length of oxygen therapy (3 ± 3.4 days, rho = 0.26, *p* = 0.08), or length of hospitalization (4 ± 3.4 days, rho = 0.13, *p* = 0.38). - Statistically significant correlation between the number of affected intercostal spaces on the right and length of NIV (3 ± 3.4, Spearman’s Rho 0.318; *p* = 0.037).	- Statistically significant correlation between the number of affected intercostal spaces on the right side and the length on the oxygen therapy (days), considered a secondary endpoint - Absence of confirmation of the primary aim, i.e. the prediction of NIV length (CPAP or high-flow nasal cannula) nor an association with length of hospitalization or the clinical score m-WCAS.
Varshney et al [14].	Prospective cross-sectional study	94 patients with signs of a respiratory tract infection (rhinorrhea and/or cough) and wheeze, median age of 11.1 months and	- Pathological LUS (≥1 finding): ≥3 B-lines per intercostal space, consolidation, pleural line abnormalities or fluid	- Good reliability between novice and expert sonologist: Kappa statistic 0.68 (95% CI 0.54 to 0.82) for a positive LUS (≥1 positive findings). Good to almost perfect agreement between raters for each finding: kappa statistic 0.88 (95% CI 0.78 to 0.98) for ≥ 3 B-lines per intercostal space, 0.62 (95% CI 0.42 to 0.82) for small consolidations, 0.88 (95% CI 0.66 to 1.00) for large consolidations and 0.55 (95% CI 0.26 to 0.84) for pleural abnormalities. - Proportion of positive LUS, along with their diagnostic accuracy (sensitivity, specificity), for children with bronchiolitis, asthma, pneumonia and asthma/pneumonia: 46% (45.8%, 72.7%), 0% (0%, 51.3%), 100% (100%, 61.1%), 50% (50%, 58.9%), respectively. - Positive LUS in 46% (33/72) of patients with bronchiolitis, 0% (0/14) of patients with asthma, 100% (4/4) of patients with pneumonia and in 50% (2/4) of those with concomitant asthma and pneumonia when categorised by final diagnosis.	- Possibility to rule out the diagnosis of asthma in case of positive LUS that could direct the clinician toward a diagnosis of pneumonia (100% of patients) or bronchiolitis (46% of patients)
Biagi et al [15].	Prospective study	87 children with a diagnosis bronchiolitis that undergone CXR because of a suspicion of concomitant pneumonia	- Subpleural consolidations, absence of air bronchograms, pleural line abnormalities, single or confluent B lines - Higher number of consolidations in the posterior lung areas	- Sensitivity and specificity of LUS for the diagnosis of pneumonia 100% and 83.9% respectively, vs. sensitivity and specificity of CXR of 96% and 87.1%. When only consolidation > 1cm was considered consistent with pneumonia, the specificity of LUS increased to 98.4% and the sensitivity decreased to 80.0%, - Strong correlation between CXR and LUS in diagnosing bacterial pneumonia (rs = 0.638, *p* < 0.0001). Stronger correlation when only consolidation size > 1 cm was considered positive for pneumonia (rs = 0.684, *p* < 0.0001). - No strong correlation between positive LUS (consolidations with bronchograms) and clinical/laboratory data (fever > 38 °C, SatO2 < 92%, WBC > 15,000/mmc, CRP > 4mg/dL), probably for the impossibility to clearly differentiate bacterial from viral disease and to predict severity of paediatric pneumonia. - Weak positive correlation between positive LUS and SatO2 < 92%, CRP > 4mg/dL and TC > 38 °C when all consolidations with bronchograms were included in the LUS positive findings. - Identification of all the cases of bronchiolitis with concomitant bacterial pneumonia with LUS.	- Higher sensitivity of LUS for the diagnosis of pneumonia than CXR (100% vs. 96%) and its specificity can reach 98.4% when only consolidations > 1 cm are considered

## Abbreviations

AIS	Alveolar Interstitial Syndrome
BTS	British Thoracic Society
CPAP	Continuous Positive Airway Pressure
CRP	C-Reactive Protein
CXR	Chest X-rays
HSJD	Hospital Sant Joan de Deu
ICU	Intensive Care Unit
LRTI	Lower Respiratory Tract Infection
LUS	Lung Ultrasound
LUS-Sc	Lung Ultrasound Score
MeSH	Medical Subject Headings
mWCAS	Modified Wood Score
NIV	Non-Invasive Ventilation
OR	Odds Ratio
PAs	Pleural line Anomalies
PED	Pediatric Emergency Department
PICU	Pediatric Intensive Care Unit
PO2	Oxygen Partial Pressure
PRISMA	Preferred Reporting Items for Systematic Review and Meta-Analyses
RSV	Respiratory Syncytial Virus
SatO2	Oxygen Saturation
SCs	Subpleural Consolidations
TC	Body temperature
TP	True Positive
US	Ultrasound
WBC	White Blood Cells
WDFM	Modified Wood Downes Ferres Score
WDFS	Wood Downes Ferres Score
